# Kisspeptin Receptor GPR54 Promotes Adipocyte Differentiation and Fat Accumulation in Mice

**DOI:** 10.3389/fphys.2018.00209

**Published:** 2018-03-13

**Authors:** Tongtong Wang, Xueqin Cui, Ling Xie, Roumei Xing, Panpan You, Yongliang Zhao, Yiqing Yang, Yongqian Xu, Li Zeng, Huaqing Chen, Mingyao Liu

**Affiliations:** ^1^Shanghai Key Laboratory of Regulatory Biology, Institute of Biomedical Sciences, School of Life Sciences, East China Normal University, Shanghai, China; ^2^Bioray Laboratories Incorporation, Shanghai, China; ^3^Department of Molecular and Cellular Medicine, Institute of Biosciences and Technology, Texas A&M University Health Science Center, Houston, TX, United States

**Keywords:** GPR54, energy metabolism, obesity, adipocyte differentiation, MAP kinase

## Abstract

GPR54, Kisspeptin-1 receptor (KISS1R), a member of rhodopsin family, plays a critical role in puberty development and has been proposed to be involved in regulation of energy metabolism. This study aims to explore the function of GPR54 in adipogenesis, lipid metabolism, and obesity in addition to its effect through hormones. Results showed that when fed a high-fat diet, the weight growth of castrated or ovariectomized *Gpr54*^−/−^ mice was significantly slower than that of WT control, together with a lower triglyceride concentration. The ratio of white adipose tissue was lower, and average size of adipocytes was smaller in *Gpr54*^−/−^ mice. Meanwhile, there were less adipose tissue macrophages (ATMs), especially pro-inflammatory macrophages. Expression of inflammatory related genes also indicated that inflammatory response caused by obesity was not as drastic in *Gpr54*^−/−^ mice as in WT mice. Liver triglyceride in *Gpr54*^−/−^ mice was reduced, especially in female mice. On the other hand, oil drop formation was accelerated when hepatocytes were stimulated by kisspeptin-10 (Kp-10). Primary mesenchymal stem cells (MSCs) of *Gpr54*^−/−^ mice were less likely to differentiate into adipocytes. When stimulated by Kp-10, 3T3-L1 cell differentiation into adipocytes was accelerated and triglyceride synthesis was significantly promoted. These data indicated that GPR54 could affect obesity development by promoting adipocyte differentiation and triglyceride accumulation. To further elucidate the mechanism, genes related to lipid metabolism were analyzed. The expression of genes involved in lipid synthesis including PPARγ, ACC1, ADIPO, and FAS was significantly changed in *Gpr54*^−/−^ mice. Among them PPARγ which also participate in adipocyte differentiation displayed a marked reduction. Moreover, phosphorylation of ERK, which involved in GPR54 signaling, was significantly decreased in *Gpr54*^−/−^ mice, suggesting that GPR54 may promote lipid synthesis and obesity development by activating MAP kinase pathway. Therefore, in addition to the involvement in hormone regulation, our study demonstrated that GPR54 directly participates in obesity development by promoting adipocyte differentiation and fat accumulation. This provided evidence of involvement of GPR54 in lipid metabolism, and revealed new potentials for the identification and development of novel drug targets for metabolic diseases.

## Introduction

Nowadays, obesity has become a global burden that seriously impairs the health and quality of people's life. Overweight or obesity is associated with abnormal fat accumulation, which is caused by imbalanced energy metabolism. Energy homeostasis is a complicated system involving many aspects of glucose or lipid metabolism including lipogenesis and lipolysis, glycolysis, and gluconeogenesis. When energy intake exceeds energy expenditure, the surplus energy will be stored as lipid and the body will become overweight. In addition to life style and eating habit, genetic factors such as mutations in genes like *ob* (Farooqi et al., 1998) and *MC4R* (You et al., 2016) were found to be involved in some of obesity development. More new genes related to obesity were identified (Locke et al., 2015; Liu et al., 2017). Chronic metabolic disturbance is directly associated with increasing prevalence of metabolic diseases including diabetes and cardiovascular diseases. It is urgent to identify key players in the metabolic pathways in order to develop potential targets that can be utilize as candidates for obesity treatment.

*Kiss1* gene was identified when investigating metastasis of human melanoma cells (Lee et al., 1996). The product of *Kiss1*, kisspeptin, is a 54-aa peptide also known as metastin. It could split into smaller peptides including kisspeptin-10 (Kp-10), Kp-13, and Kp-14. The peptide share similar functions and activities. The specific receptor of kisspeptins, GPR54, belongs to the rhodopsin family (Ohtaki et al., 2001). The critical function of GPR54/KISS1 system in nervous-gonadal axis was first reported in 2003 (de Roux et al., 2003). Since then, more studies have demonstrated that GPR54/KISS1 plays an important role in puberty development (Gutiérrez-Pascual et al., 2007; Kauffman et al., 2007; Sonigo and Binart, 2012). Interestingly, in addition to high expression in pituitary gland and placenta (Kotani et al., 2001), GPR54 is also expressed in pancreas, brain, liver, and skeletal muscle (Kalamatianos et al., 2008). This indicates that GPR54 also play roles in other biological processes, especially in energy metabolism. Previous studies on GPR54/KISS1's function in metabolic system were mostly performed *in vitro*. Studies were focused on demonstrating differential expression of *Kiss1* gene in adipose tissue or hypothalamus under different situations or stimulations in an effort to show the potential involvement of *Kiss1* in energy metabolism. The results, however, were inconclusive. For example, adult rats showed a reduction in *Kiss1* mRNA and a decreased luteinizing hormone after fasting (Castellano et al., 2005; Brown et al., 2008). GPR54 expression was reduced in adult rats when fasted for 12–48 h, but increased in pubertal rats when fasted for 72 h (Castellano et al., 2005; Luque et al., 2007). Early in 1996, *Kiss1* gene was found highly expressed in pancreas and implied kisspeptins involvement in pancreativ activity (Lee et al., 1996). A series of studies showed that kisspeptins and their receptor co-localized in alpha and beta cells of pancreas (Hauge-Evans et al., 2006). It was also shown that Kp-54 and Kp-10 could stimulate primary pancreas cells to secret insulin (Hauge-Evans et al., 2006; Wahab et al., 2011). Yet, other studies reported that kisspeptins inhibited insulin secretion in a concentration dependent manner (Bowe et al., 2009; Vikman and Ahren, 2009). Data from *in vivo* studies were limited. A recent report stated that *Gpr54*^−/−^ female mice displayed a heavier body weight and an impaired glucose tolerance (Tolson et al., 2014). On the contrary, another paper in the same year reported inhibition of *Kiss1* attenuated hyperglycemia. Increased kisspeptins were detected in both liver and serum of type 2 diabetes patients and mice (Song et al., 2014). This kind of variations in data from *in vitro* as well as *in vivo* studies implied that GPR54/KISS1 signaling might play a complex role, exerting different functions at different tissues, different stages, and under different biological conditions. Further investigations need to be carried out to help elucidating and understanding the different functions and mechanisms of GPR54/KISS1 signaling. In this study we mainly used castrated or ovariectomized *Gpr54*^−/−^ mice in order to minimize hormone effects, and evaluated the direct functions of GPR54 in lipid metabolism, and the possible mechanism for the signaling pathway in adipose tissue. Results showed that GPR54 deficiency in mice led to slower body weight increase and reduced adipocyte differentiation. In *in vitro* cell system, GPR54/KISS1 activation led to enhanced adipocyte differentiation and triglyceride accumulation, suggesting involvement of GPR54/KISS1 signaling in adipogenesis, lipid metabolism, and obesity development, through a mechanism independent of sex hormone signaling.

## Materials and methods

### Animals

GPR54 deficient C57BL/6 mice were obtained from Dr. Eric L. Gustafson at Schering-Plow Research Institute (Kenilworth, NJ, USA). Genotyping was conducted by PCR as described previously (Funes et al., 2003). The genotypes of all animals were verified as correct (Supplementary Figure 1). Mice were bred and maintained at 4–5 per cage, in a constant room temperature (25°C) and photoperiod cycle (12 h light/12 h dark) in specific pathogen-free (SPF) level Laboratory at Animal Center of East China Normal University, with free access to water and food. To minimize hormone influence, mice were castrated (female ovariectomized) at 20–21 post-natal days under avertin anesthetization. This study was carried out in accordance with the recommendations of NIH guidelines. All protocols were approved by the institutional Animal Ethics Committee (permit No. m20140704).

### Histological analysis of adipose tissues

Mice were euthanized by cervical dislocation. Adipose tissue was excised and fixed overnight in 4% paraformaldehyde and embedded in paraffin. Sections at 4 μm were cut and stained by Hematoxylin and eosin (HE) for histological analysis. Digital images (200×) were captured from five random field per section using a Leica microscope, then analyzed using ImageJ software. To evaluate adipocyte size, a minimum of 100 independent mature adipocytes per mouse was measured. Number of adipocytes was assessed from at least three samples of three mouse.

### Flow cytometry analysis of adipose tissue macrophages (ATMs)

Inguinal adipose tissue was dissected and lymph nodes were removed. Adipocytes and stromal vascular cells were separated by conventional method using type II collagenase (Sigma-Aldrich) digestion (Cho et al., 2014; Kitada et al., 2016). Antibodies were purchased from Biolegend, including APC anti-mouse F4/80 monoclonal antibody (Clone BM8, Cat. No. 123115), FITC anti-mouse CD206 antibody (Clone C068C2, Cat. No. 141703). Analysis of ATMs was first gated on living cells, followed by analysis for F4/80 and CD206 by flow cytometry (FACSCalibur, Becton Dickinson, USA).

### RT-PCR and real-time PCR analysis

Total RNA was isolated from homogenated tissues using Trizol (Invitrogen). cDNA was synthesized using PrimeScript™ RT kit (Takara). RT-PCR products were analyzed on 1.5% agarose gel. Real-time PCR was conducted using SYBR Green PCR Master Mix (Takara) according to the manufacturer's instruction and run on real-time PCR system (MX3005p, Stratagene, USA) and analyzed using MXProv 4.1. Primers (listed in Supplementary Table 1) were synthesized by Shanghai Biosune (Shanghai, China).

### Adipocyte differentiation models

3T3-L1 system: mouse 3T3-L1 preadipocytes (from The National Center for Drug Screening, Shanghai, China) were maintained in complete high-glucose Dulbecco's modified Eagle's medium (DMEM, GIBCO, USA) supplemented with 10% fetal bovine serum (FBS) at 37°C, in a humidified incubator with 5% CO_2_. For induction of adipose differentiation, 30,000 cells in 300 ml per well were seeded in 48-well plates and incubated until confluence. After incubated for another day, cells were exposed to inducer A (complete medium with 0.5 mM isobutylmethylxanthine, 1 μM dexamethasone, 0.2 mM indometacin, and 10 μg/ml insulin). After 3 days, the medium was changed to inducer B (complete medium containing 10 μg/ml insulin) and incubated for another 3 days. After that cells were cultured in complete medium for 2 days. As for Kp-10 stimulation, different concentrations of Kp-10 were added together with inducer A and B, with DMSO as control. Insulin-dependent glucose uptake experiment was conducted as usual. Briefly, after induction, 3T3-L1 cells were starved then stimulated with 0.6 μg/ml insulin for 30 min. The uptake of the fluorescent glucose homolog 2-NBDG (Invitrogen) was measured by incubation cells with 80 nM 2-NBDG for 15 min. After wash, fluorescence was measured on a FLUOstar Omega (BMG LABTECH).

Mesenchymal stem cell (MSC) system: MSCs were separated from bone marrow. Femurs and tibiae were removed after mice were euthanized. All connective tissue were cleaned and the ends of each tibia and femur were clipped. The marrow was blown into a 10 cm dish by a 5 ml syringe, and cultured in 8 ml complete high-glucose DMEM medium for overnight. Non-adherent cells were removed and adherent cells were washed. Fresh complete medium was then added and cultured for 2–4 days. The cells were lifted by incubation with trypsin/EDTA for 2–3 min, plated at 100,000 cells per well in 48-well plates. Induction for adipose differentiation and Kp-10 stimulation was conducted as described in 3T3-L1 system.

Kp-10 peptide was synthesized according to reference (Asami et al., 2012) in which analog 13 showed not only high metabolic stability but also excellent GPR54 agonistic activity to human and mouse.

### The cellular non-alcoholic fatty liver disease (NAFLD) model

The cellular steatosis model suitable for investigation of the impact of GPR54 on fat accumulation in liver was established in a human normal liver cell line LO_2_. LO_2_ cells were purchased from the Cell Bank of Type Culture Collection of Chinese Academy of Science (Shanghai, China), and cultured in complete high-glucose DMEM medium. For steatosis induction, cells were exposed to a mixture of free fatty acids (0.25 mM sodium oleate and 0.125 mM palmitate) for 12 h. Different concentrations of Kp-10 were added together with the free fatty acids, with DMSO as normal control.

### Immunoblotting

Adipose tissue was homogenated in Ripa lysis buffer (with 1mM PMSF, Merck Millipore). Proteins were extracted and separated on 10% SDS-PAGE gel, and electroblotted onto a nitrocellulose membrane (Schleicher and Schuell MicroScience) using a Mini trans-blot apparatus (Bio-Rad). The membrane was blocked with 5% skim milk-PBS (pH 7.4) for 1 h at room temperature, then incubated with primary antibody for overnight at 4°C. After washed in PBS-1%0 Tween-20, the membrane was incubated in secondary antibody for 2 h at room temperature, then analyzed on the Odyssey infrared imaging system (LI-COR). All the antibodies were purchased from Cell Signaling Technology except rabbit anti-PPARγ polyclonal antibody (Proteintech, USA, Cat. No. 16643-1-AP, Dilution: 1:1,000). These included P44/42 MAPK (Erk1/2) antibody (Cat. No. 9102, Dilution: 1:1,000), Phospho-p44/42 MAPK (Erk 1/2) monoclonal antibody (Clone 197G2, Cat. No. 4377, Dilution: 1:1,000), p38 MAPK rabbit polyclonal antibody (Cat. No. 9212, Dilution: 1:1,000) and phospho-p38 MAPK rabbit monoclonal antibody (Clone D3F9, Cat. No. 9211, Dilution: 1:1,000).

### Statistics

All data were presented as mean ± SD. Statistical analysis was performed using Graphpad Prism. Student's *t*-test was used for comparison of two groups and one way ANOVA was used for multiple comparison. *p*-value < 0.05 were considered as statistically significant.

## Results

### HFD induction enhanced GPR54 expression in adipose tissues

**Figure 1:**
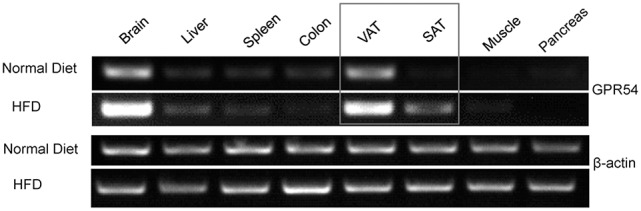
GPR54 mRNA level in metabolism related tissues. RT-PCR analysis for GPR54 in different tissues of 10-week old WT mice fed on normal diet or HFD. Total RNA was extracted using TRIzol. β-actin was used as control.

In order to explore the possibility of GPR54 involvement in metabolism, GPR54 expression profile in metabolism related tissues was assessed by RT-PCR. Data showed that GPR54 was highly expressed in brain and visceral adipose tissue (VAT). Moreover, GPR54 expression increased significantly in brain, VAT as well as subcutaneous adipose tissues (SAT) of HFD-fed mice (Figure 1). These data suggested that, in addition to the involvement in central nervous system, GPR54 may play a direct role in adipose tissues and participate in lipid metabolism.

### Effect of GPR54 on obesity development

**Figure 2:**
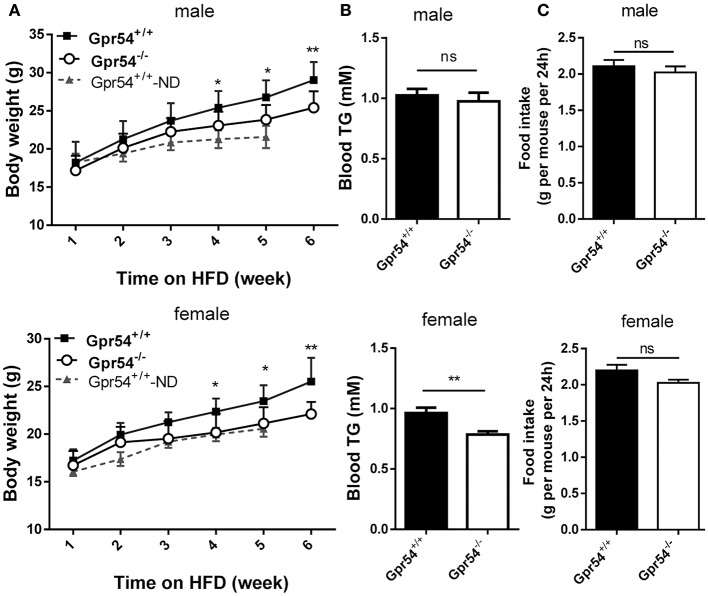
Effect of GPR54 deficiency on body weight, blood TG and food intake after HFD induction. **(A)** Body weight after HFD induction, compare with normal diet (ND)-fed WT mice. **(B)** Blood TG and **(C)** food intake of WT and *Gpr54*^−/−^ mice. Data were expressed as mean ± *SD* (*n* = 10–12), ^*^p < 0.05, ^**^p < 0.01.

To study the direct function of GPR54 on metabolism, *Gpr54*^+/+^ (WT) and *Gpr54*^−/−^ mice were castrated or ovariectomized at 20–21 post-natal days to equalize sex hormones. Mice were maintained on normal diet until 6 weeks old, then transferred to HFD (noted as day 0) for 6 weeks. During the HFD period, consumption of water and food was measured every 2 days, and body weight was measured every week. Body weight in *Gpr54*^−/−^ mice was significantly lower than that in WT. This phenomenon was manifested in both male and female mice (Figure 2A). Meanwhile, female *Gpr54*^−/−^ mice displayed significantly lower triglyceride (TG) content in blood than female WT mice. Male mice showed the same trend but did not reach significance (Figure 2B). There was no significant difference in food intake between *Gpr54*^−/−^ and WT mice (Figure 2C), indicating that the less severe obesity induced in *Gpr54*^−/−^ mice was not a result of difference in food consumption.

### Unaltered glucose metabolism in GPR54 deficient mice

**Figure 3:**
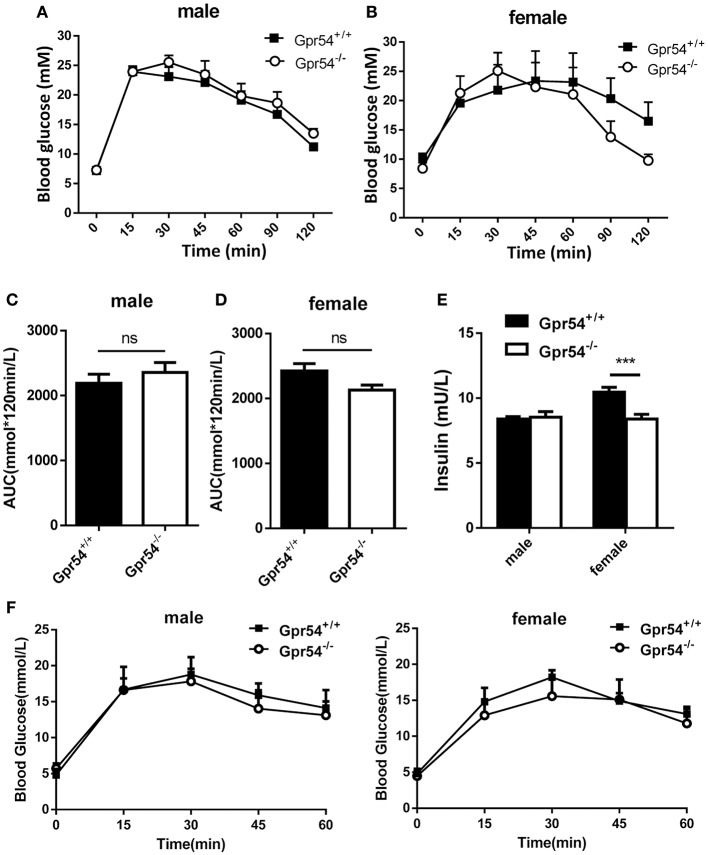
Effect of GPR54 deficiency on glucose metabolism. **(A,B)** OGTT time course and **(C,D)** respective AUC values of the HFD-fed mice (*n* = 7–10). **(E)** Insulin levels of serum from two genotypes fed on HFD. **(F)** OGTT of mice kept on normal diet (*n* = 6). Mice were fasted 12 h before the test, followed by oral glucose challenge at 2 g/kg. Data were expressed as mean ± *SD*, ^***^p < 0.001.

Obesity development could lead to insulin resistance and impaired glucose tolerance. Regulation of blood glucose in *Gpr54*^−/−^ and WT mice was examined using oral glucose tolerance test (OGTT). As shown in Figures 3A,B both WT and *Gpr54*^−/−^ mice displayed a higher than normal level of fasting blood-glucose (>7.0 mM), suggesting impaired glucose tolerance by HFD induction. This was quite different from mice kept on normal diet which showed a normal fasting blood-glucose (Figure 3F). However, *Gpr54*^−/−^ mice did not showed significant difference in glucose tolerance as compared to WT control, no matter fed on a HFD or normal diet (Figures 3A–D,F). In other hand, significant reduction of insulin was only found in female *Gpr54*^−/−^ mice (Figure 3E). Altogether, GPR54 did not play a very important role in glucose metabolism in this study.

### GPR54 regulation on adiposity and adipose tissue macrophages

**Figure 4:**
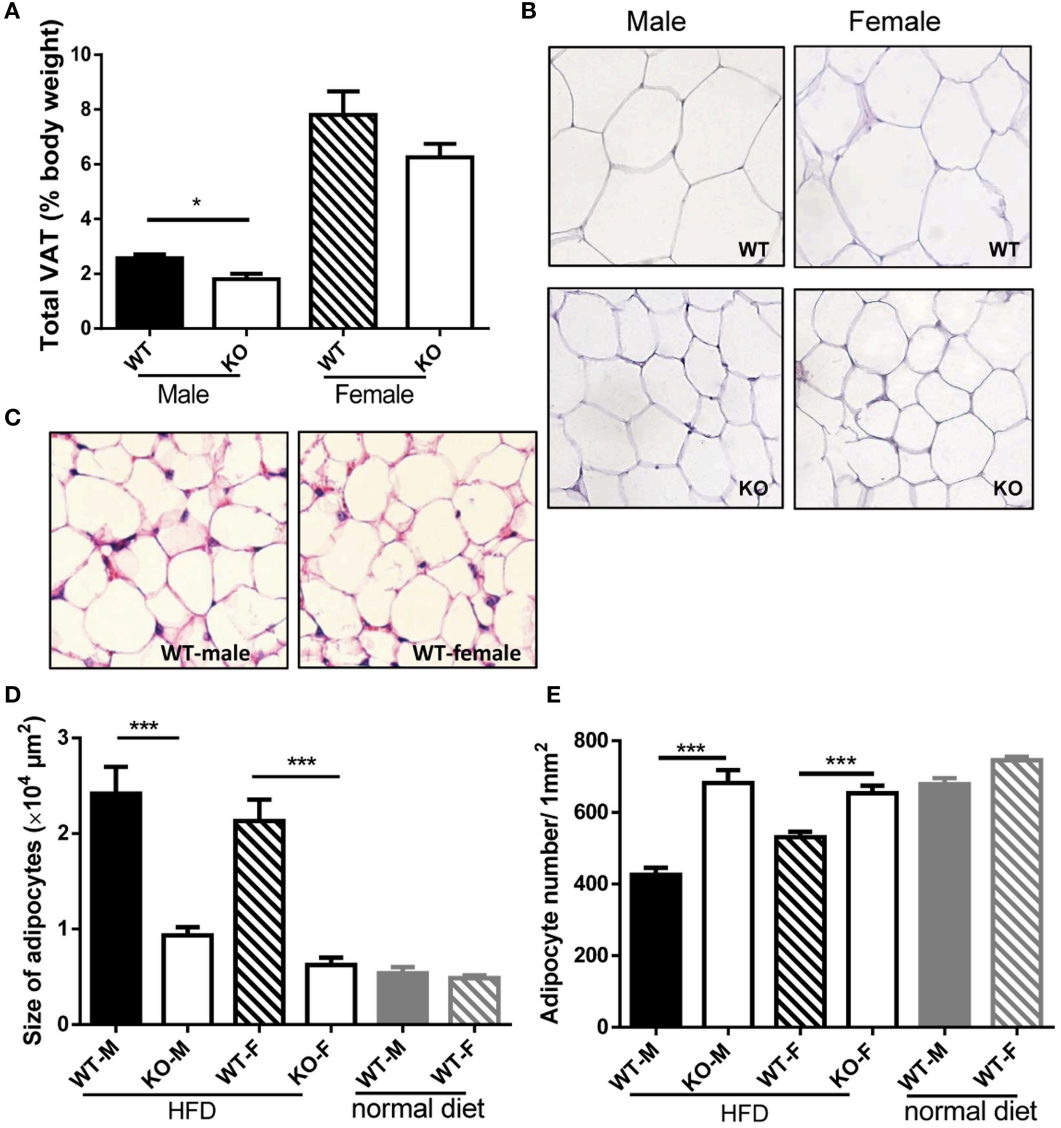
VAT percentage and HE staining of adipose tissue. **(A,B)** VAT percentage to body weight of WT (*Gpr54*^+/+^) and KO (*Gpr54*^−/−^) mice after HFD induction (*n* = 5), and representative image of adipocytes (magnification ×200). **(C)** Representative image of adipocytes from WT mice kept on normal diet. **(D,E)** Statistical results for average adipocyte size and adipocyte density. M, male; F, female. Data were expressed as mean ± *SD*, ^*^p < 0.05, ^***^p < 0.001 vs. respective WT control.

In order to investigate whether the reduced weight growth was associated with alteration in adipose tissue, we measured body fat of mice. After HFD induction, GPR54 deficient mice demonstrated significantly reduced VAT as compared to WT littermates. Male *Gpr54*^−/−^ mice displayed a significantly lower relative VAT percentage than WT, while female mice had the same trend but did not reach significance (Figure 4A). Consistent with this result, histology analysis by HE staining showed that adipocytes from both female and male *Gpr54*^−/−^ mice had smaller size and higher adipocyte density than WT control, close to the normal phenotype of adipocytes from normal diet-fed WT mice (Figures 4B–E). These data suggested that GPR54 deficiency attenuated HFD-induced adipocyte hypertrophy and lipid accumulation in mice, resulting in slower body weight growth.

**Figure 5:**
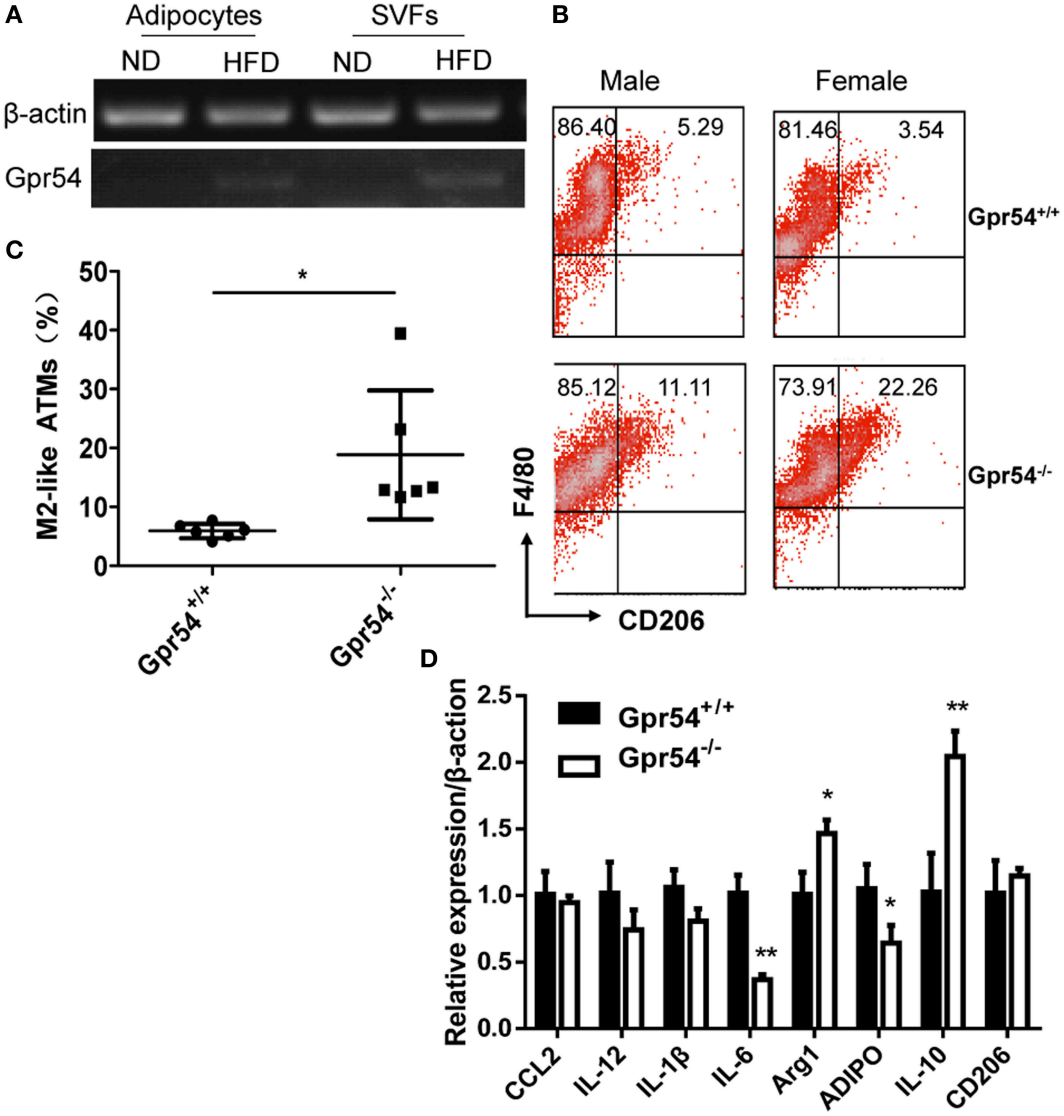
GPR54 deficiency led to alteration of ATMs in obese mice. **(A)** GPR54 mRNA expression in mature adipocytes and stromal vascular fractions (SVF) of normal diet (ND) and HFD-fed WT mice. **(B)** Representative FACS histogram for SVFs from *Gpr54*^−/−^ and WT littermates. F4/80^+^: total macrophages. CD206^+^: M2-like macrophages. **(C)** Different percentage of M2-like in total macrophages. Data were expressed as mean ± *SD* (Total six pairs of mice with three male and three female), ^*^p < 0.05. **(D)** Expression of pro-inflammatory and anti-inflammatory genes in VAT of two genotypes. Arginase 1 (Arg1); Adiponectin (ADIPO). ^**^p < 0.01.

It was reported that obesity increased ATM accumulation in visceral adipose depots, and was associated with qualitative changes of ATMs (Weisberg et al., 2003; Harman-Boehm et al., 2007). To this end ATMs in fat were analyzed. Adipocytes and stromal vascular fractions (SVF) were separated from WT mice. Data showed that GPR54 expression was increased in HFD-fed mice as compared to that in normal diet-fed mice (Figure 5A). SVFs were then separated from WT and *Gpr54*^−/−^ obese littermates. Cells were stained by F4/80 and CD206 antibody, and analyzed by FACS analysis. Results showed that *Gpr54*^−/−^ mice presented less ATMs than WT littermates, together with significantly more M2-like (F4/80^+^CD206^+^) anti-inflammatory macrophages (Figures 5B,C). In addition, a trend of reduced inflammation was also manifested in reduced expression of IL-6 and adiponectin (ADIPO), as well as increased IL-10 and arginase 1 (Arg1) in *Gpr54*^−/−^ VAT (Figure 5D). These data suggested that inflammatory response caused by obesity in *Gpr54*^−/−^ mice was not as drastic as that in WT mice.

### Effect of GPR54 deficiency on liver

**Figure 6:**
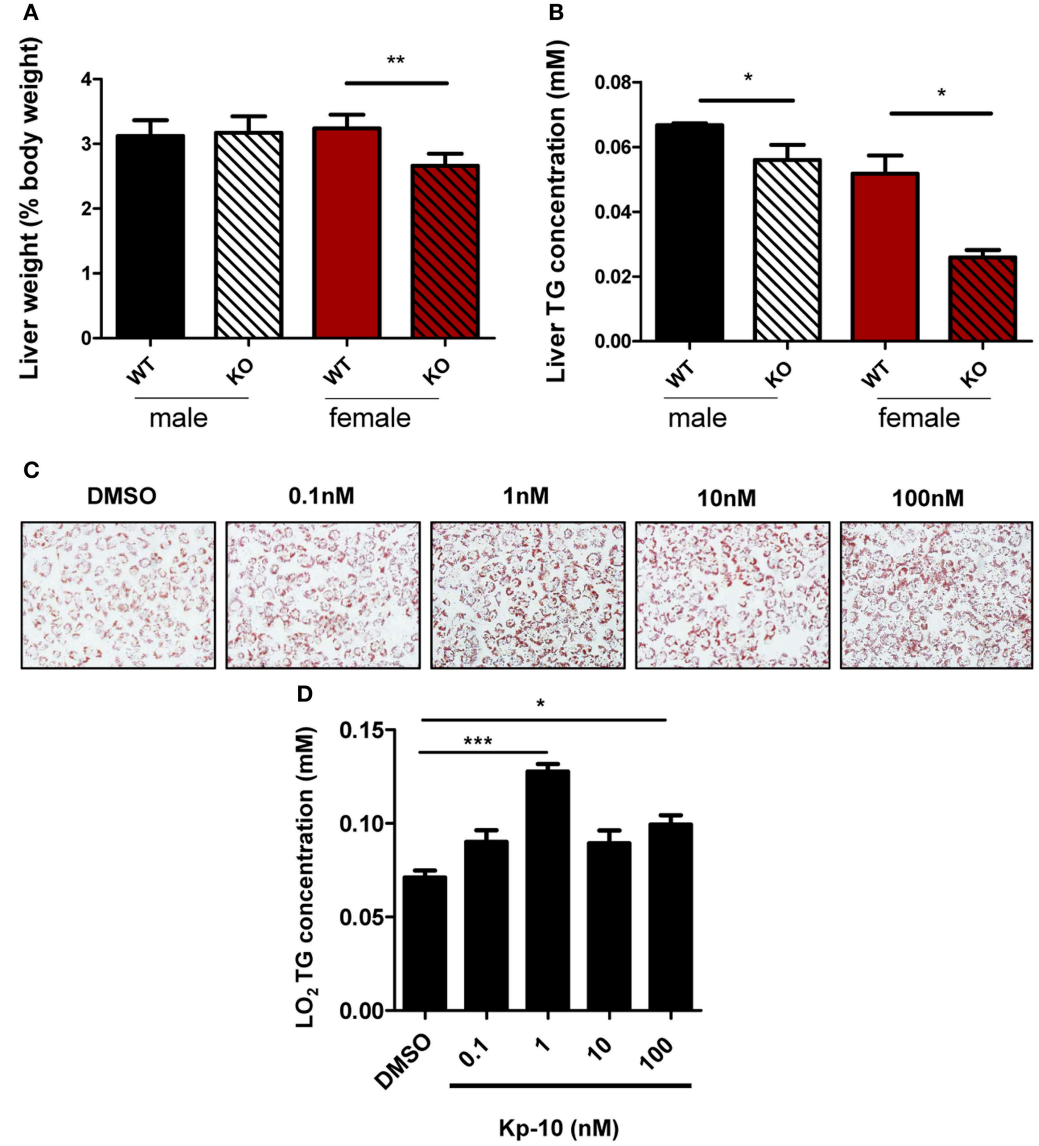
Alteration of liver in GPR54 deficient mice. **(A)** Relative liver weight (as percentage of body weight) of KO (*Gpr54*^−/−^) and WT (*Gpr54*^+/+^) littermates after HFD induction. **(B)** TG content in liver. Data were expressed as mean ± SD (*n* = 4–6), ^*^p < 0.05, ^**^p < 0.01 vs. respective WT control. **(C)** Kp-10 stimulation promoted lipid accumulation in LO2 cells. Different concentrations of Kp-10 were added in the NAFLD model of LO2 induced by free fatty acids. Cells were stained by Oil Red O solution (magnification ×200). **(D)** TG content in LO2 cells. Data were expressed as mean ± SD (*n* = 3), ^*^p < 0.05, ^***^p < 0.001 vs. DMSO control.

Livers of different groups of mice were examined. Relative liver weight in *Gpr54*^−/−^ female mice was significantly lower than that in WT littermates (Figure 6A). No significant difference in liver weight was revealed in male counterparts. TG content in *Gpr54*^−/−^ liver of both sexes was significantly reduced as compared to that in respective WT counterparts (Figure 6B).

To further confirm regulatory function of GPR54 in steatosis, normal human hepatocytes LO_2_ were used to establish the NAFLD model. The shortest kisspeptin Kp-10 stimulation accelerated adipose accumulation, with peak stimulation at 1 nM. In addition, TG synthesis in the cells was found to be enhanced by Kp-10. The peak enhancement also appeared at 1 nM (Figures 6C,D). Therefore, it can be concluded that Kp-10 stimulation promoted TG synthesis and adipose accumulation of liver cells. The pattern of Kp-10 stimulation did not show typical concentration gradient effect, which was consistent with previous report that Kp-10 may exert opposite effect at higher concentrations (Olbrich et al., 2010).

### GPR54 promoted adipocyte differentiation

**Figure 7:**
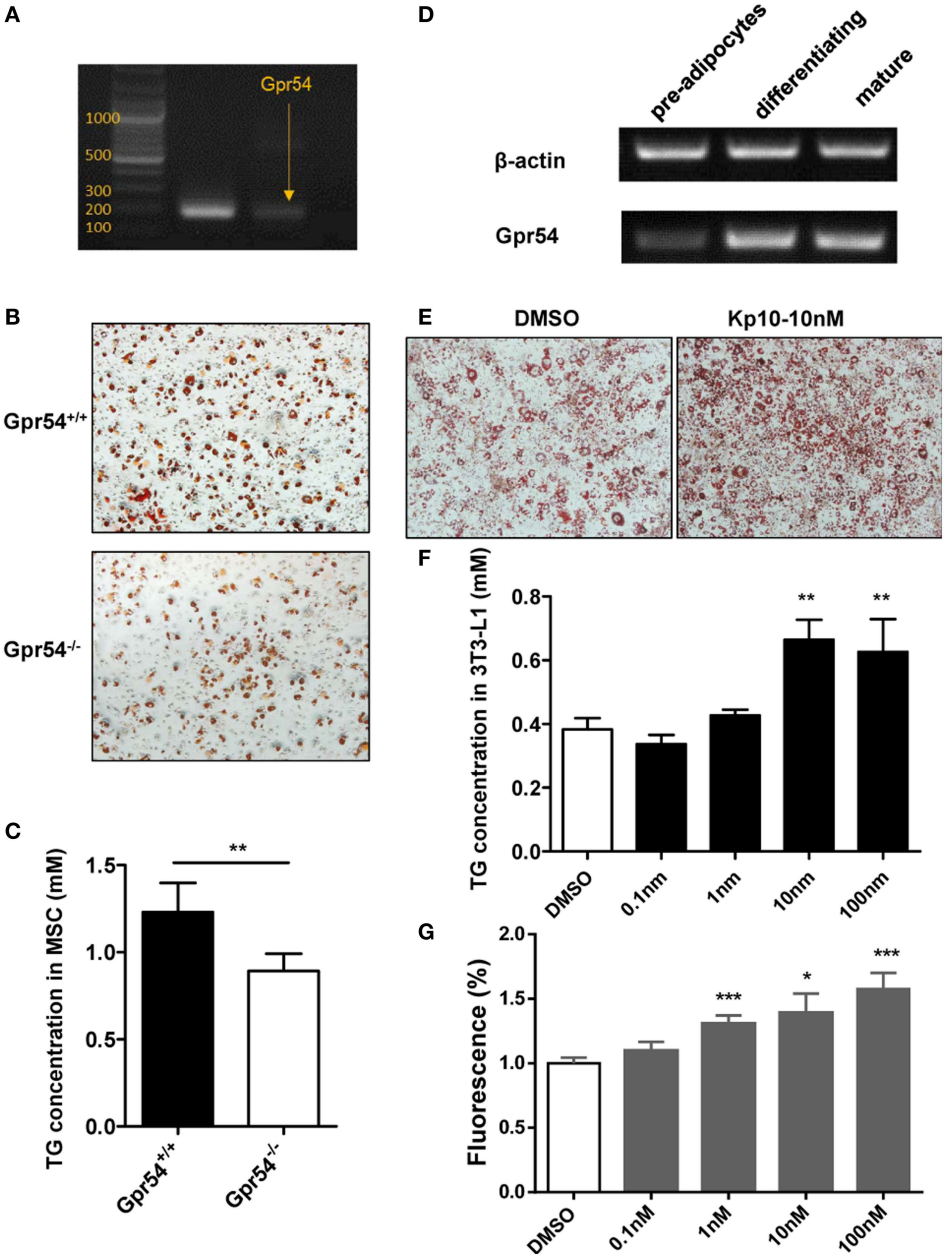
Kp-10 stimulation of GPR54 promoted adipocyte differentiation. **(A)** GPR54 expression in MSCs. **(B)** Oil Red O staining of MSC differentiation into adipocytes (magnification ×100). **(C)** Cellular TG contents of MSCs (*n* = 6). **(D)** GPR54 expression in different stages of differentiated 3T3-L1 cells. **(E)** Oil Red O staining of 3T3-L1 cells stimulated by 10 nM Kp-10 vs. DMSO control (magnification ×100). **(F)** Cellular TG of 3T3-L1 cells (*n* = 3). **(G)** Insulin-dependent glucose uptake in 3T3-L1 cells stimulated by Kp-10 (*n* = 12). Data were expressed as mean ± *SD*, ^*^p < 0.05, ^**^p < 0.01, ^***^p < 0.001 vs. DMSO control.

We next established adipocyte differentiation models with both bone marrow-derived MSCs and 3T3-L1 cells. MSCs from WT mice were found to express GPR54 (Figure 7A). When MSCs separated from three-week old *Gpr54*^−/−^ and WT mice were induced, Oil Red O staining showed that MSCs from *Gpr54*^−/−^ mice displayed a lower differentiation as compared to that from WT littermates (Figure 7B). At the same time, intracellular TG in the differentiated cells from *Gpr54*^−/−^ mice was lower than that from WT mice (Figure 7C).

The above results were confirmed in 3T3-L1 system in which GPR54 mRNA expression displayed an upregulated trend in the differentiation process (Figure 7D). In this system, Kp-10 stimulation promoted adipose differentiation (Figure 7E), accompanied by the increased TG in cells (Figure 7F). In addition, Kp-10 stimulation enhanced insulin-dependent glucose uptake in 3T3-L1 cells (Figure 7G). Altogether, these data clearly demonstrated that Kp-10 stimulation of GPR54 promoted adipocyte differentiation and metabolic function.

### Mechanism of GPR54 regulation of adipocyte differentiation

**Figure 8:**
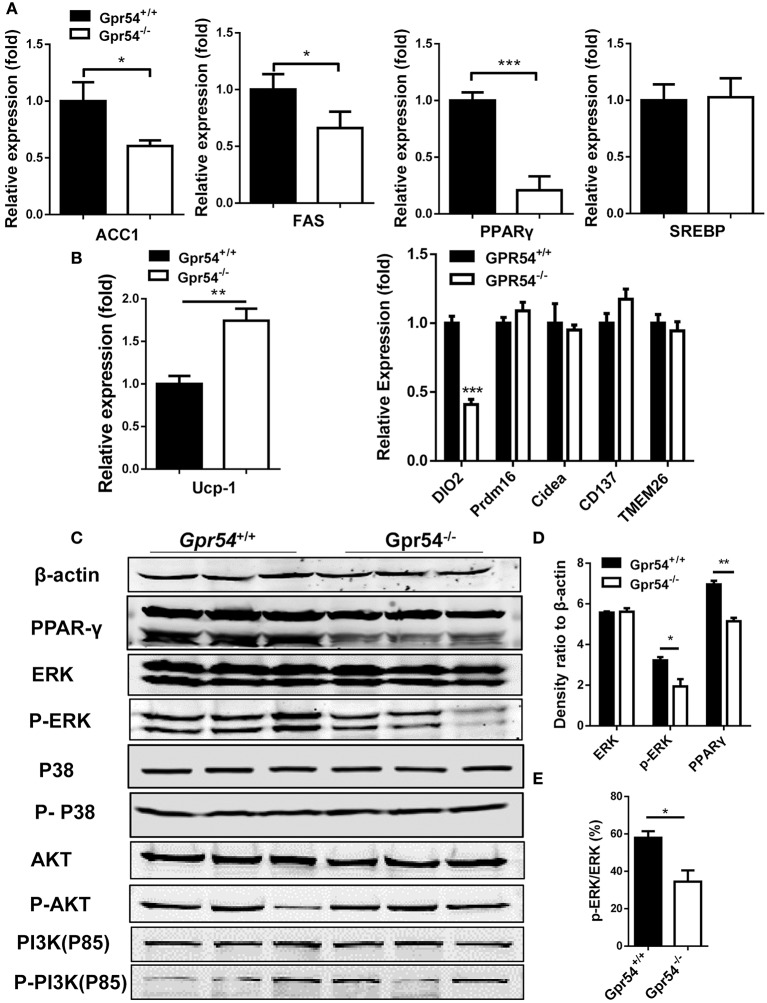
GPR54 regulatory effects on key genes related to lipid metabolism. **(A)** Expression of key genes in white adipose tissue of HFD-induced mice, analyzed by real-time PCR analysis (triplicates). ACC1, acetyl-CoA carboxylase 1; FAS, fatty acid synthase; SREBP, sterol regulatory element-binding protein; PPAR, peroxisome proliferator activated receptor; **(B)** Expression of browning genes. UCP, uncoupling protein; PRDM16, PR domain containing 16. **(C)** Effects of GPR54 on PPARγ, MAPK, and AKT/PI3K pathways in adipose tissue of HFD-induced mice. **(D)** Density ratio of different genes. **(E)** Percentage of phosphorylated ERK. PI3K, Phosphatidylinositol 3-kinase. Data were expressed as mean ± *SD*, ^*^p < 0.05, ^**^p < 0.01, ^***^p < 0.001 vs. WT control.

In order to gain more insight into GPR54 signaling in lipogenesis and lipid metabolism, key lipid metabolic genes including FAS and ACC1 were assessed in experimental *Gpr54*^−/−^ and WT littermates by real-time PCR analysis. In adipose tissue of *Gpr54*^−/−^ mice, reduction was shown in adipogenic markers such as FAS, ACC1, and PPARγ, with PPARγ presenting the largest reduction (one-fifth). No change was detected in the level of SREBP (Figure 8A). UCP-1 expression was increased but no significant increase was found in other browning related genes. DIO2 even displayed a marked reduction (Figure 8B). These data suggest that there was no browning effect associated with GPR54 deficiency. In addition, mRNA level of the lipid droplet-associated protein, perilipin (PLIN), was analyzed and significant reduction was found in PLIN1 of liver and PLIN3 of adipose tissue of GPR54 deficient mice (Supplementary Figure 2).

The marked alteration in PPARγ prompted us to analyze its protein level, as it is a master regulator of adipocyte differentiation (Ahmadian et al., 2013). Reduction of PPARγ expression in GPR54 deficient mice was confirmed in Western blotting using adipose tissue extracts. It was previously demonstrated that mitogen-activated protein kinases (MAPK) p38 and ERK1/2 were markers of GPR54 activation (Castaño et al., 2009; Cvetkovic et al., 2013). Moreover, inhibition of MAPK/ERK signaling pathway could suppress adipogenesis and down regulate PPARγ expression (Wang et al., 2009). We proposed that MAPK might be a convergence point for GPR54 signaling and adipogenesis signaling. By checking these MAP kinases in adipose tissues, western blot analysis was carried out. Data showed a, reduced phosphorylation of ERK (Figures 8C–E). In addition, insulin responsiveness of adipocytes is important, Akt-PI3K signaling pathway, and no significant alteration was found in expression and phosphorylation of AKT or PI3K (Figure 8C). Therefore, it was concluded that GPR54 deficiency inhibited ERK phosphorylation and reduced PPARγ expression.

## Discussion

This study investigated functions of GPR54 on lipid metabolism. GPR54 deficiency substantially attenuated body weight gain and TG level in HFD-induced castrated/ovariectomized mice. These findings were consistent with the observation in adipose tissue, as GPR54 deficient mice presented a reduced adipose tissue percentage and smaller adipocyte size, as well as reduced pro-inflammatory ATMs. Meanwhile, GPR54 agonist Kp-10 upregulated TG synthesis and lipid accumulation in normal liver cells, as well as increased TG synthesis and adipose differentiation in both MSC and 3T3-L1 systems. These results suggested that anti-obesity effect of GPR54 deletion in HFD-fed mice was mediated via inhibition of adipocyte differentiation and lipogenesis, reducing serum TG level by altering hepatic lipid metabolism.

GPR54 is highly expressed in placental tissue and central nervous system, and expression had also been reported in peripheral tissues such as adipose, thymus (Kotani et al., 2001; Muir et al., 2001; Funes et al., 2003; Herbison et al., 2010) and peripheral blood lymphocytes (Xing et al., 2018). Here we manifested GPR54 expression in adipose tissues which was markedly increased in HFD-induced mice. Increased expression was also found in mature adipocytes and SVFs. In addition, GPR54 expression was exhibited in MSCs and 3T3-L1 pre-adipocytes, and expression increased when cells were induced and differentiated into mature adipocytes. The wide distribution in adipocytes and adipose tissues suggest that GPR54 plays a direct role in lipid metabolism. Since GPR54/KISS1 system is crucial in puberty development and sex hormone secretions which have effects on lipid metabolism and body weight (Butera, 2010; Lizcano and Guzman, 2014). We used castrated or ovariectomized WT and *Gpr54*^−/−^ mice in order to equalize sex hormones. While intact *Gpr54*^−/−^ mice showed an increased body weight as compared to WT partly due to reduced sex hormones (data not shown), we found quite different phenotypes in castrated or ovariectomized mice. Our result indicate that in addition to its involvement in reproduction, GPR54 could influence body weight growth and lipid metabolism through a pathway that was independent of sex steroids.

Although no GPR54 expression was reported in macrophages, ATMs are predominant leukocytes in fat and key contributors to obesity associated inflammation. Previous observations supported the general model that ATMs undergo a phenotypic switch from an anti-inflammatory M2 state to a pro-inflammatory M1 state when animals develop obesity (Lumeng et al., 2007; Chawla et al., 2011). In our experiment, the phenotype of ATM polarization was consistent with overall results including body weight and VAT percentage. Less M1-type and more M2-type macrophages presented in less obese *Gpr54*^−/−^ mice as compared to WT.

Many studies on GPR54/KISS1 expression related to energy metabolism had been carried out which mainly via *in vitro* systems. Results were unclear and hard to reach a conclusion (Hauge-Evans et al., 2006; Luque et al., 2007; Brown et al., 2008; Vikman and Ahren, 2009). Only a few reports about GPR54 deficiency on energy metabolism were available. Earlier data came from studies regarding hypogonadism associated with *Kiss1* or *Gpr54* knockout mice. It was shown that GPR54 or KISS1 deficient mice displayed almost no body weight difference except that GPR54 deficient males weighted slightly less than WT males (Lapatto et al., 2007). Recently a report in JCI stated that *Gpr54* knockout mice displayed a sexually-dimorphic metabolic phenotype, with male *Gpr54*^−/−^ showed no alteration, but normal diet fed female *Gpr54*^−/−^ mice ovariectomized at two weeks old developed obesity by 4–5 months of age. In addition, female *Gpr54*^−/−^ mice ovariectomized at adulthood then directly transferred to HFD also displayed a mildly enhanced body weight increase (Tolson et al., 2014). However, they reported glucose intolerance in normal diet-fed *Gpr54*^−/−^ mice, but not in HFD-induced *Gpr54*^−/−^ mice. Also it was very difficult to explain the huge difference between male and female mice. Moreover, no obesity phenotype was reported in studies on human subjects with *Gpr54* or *Kiss1* mutations (de Roux et al., 2003; Seminara et al., 2003; Topaloglu et al., 2012). Later in young animals (6 weeks old), it was found that female *Gpr54*^−/−^ mice fed on normal diet had normal body weights, normal feeding and glucose tolerance as compared to female WT, although some alteration in adiposity was exhibited (Tolson et al., 2016). Another study in Cell Metabolism reported that inhibition to *Kiss1* led to attenuated hyperglycemia but no change in body weight. Higher kisspeptin levels in diabetic patients as well as diabetic/obesity mice were observed, while kisspeptin knockdown led to insulin secretion and improved glucose intolerance (Song et al., 2014). Their work in some way agreed with our result, but they emphasized more on effect of glucose metabolism of liver kisspeptin, while our study investigated effect of GPR54/KiSS1 system on lipid metabolism, mostly in adipose tissues. Both observed that inhibition of GPR54/KiSS1 signaling could prevent obesity or diabetes. Variations in above study results may come from different experiment design. For example the JCI paper kept their ovariectomized (at 2 weeks) mice in normal diet, whereas in our experiment, we castrated mice at 3 weeks then accustomed them in normal diet for 3 weeks before transferred to HFD. On the other hand these variations may be a reflection of the complex roles of GPR54/KISS1 pathway, including distinctive regulatory function at different ages of the animals and under different situations. It was reported that KISS1 had varying roles across the reproductive lifespan including conception, puberty, menopause, and aging (Clarke and Dhillo, 2016).

According to the expression profile, the regulatory mechanisms of GPR54 might reflect GPR54 signaling impairment in the brain or adipose tissues. Kisspeptin neurons in brain were reported to regulate POMC and NPY neurons (Backholer et al., 2010). However, no significant change in food intake was observed in our *Gpr54*^−/−^ mice, indicating there might be other pathways involved, possibly through dysregulated GPR54 signaling in adipose tissues. As some of the metabolic phenotypes in *Gpr54*^−/−^ mice were more prominent in females than in males in our experiment, the preliminary study for the regulatory mechanism of GPR54 in adipose tissue was conducted in female mice. ERK and p-38 activation have been previously established as part of GPR54 signaling (Castaño et al., 2009; Cvetkovic et al., 2013), On the other hand, inhibition of MAPK/ERK signaling pathway was reported to suppress adipogenesis and down regulation of PPARγ (Wang et al., 2009). Our result confirmed that deficiency of GPR54 signaling in adipose tissue led to inhibition of ERK phosphorylation, suggesting that GPR54 signaling may converge with lipogenesis signaling at the level of MAPK. The inhibited MAPK activation probably lead to reduction in PPARγ expression and adipogensis.

In conclusion, we demonstrated that GPR54 played a direct pro-obesity role by promoting adipocyte differentiation and lipid accumulation in addition to its previously established role in reproductive regulation. These results help to elucidate the complex biological functions of GPR54/KISS1 system, and will be beneficial for further identification and development of potential drug targets for metabolic diseases.

## Author contributions

TW, HC, and ML: Conceived the experiments; TW, XC, LX, RX, PY, YZ, YY, and YX: Conducted the experiments; HC, LZ, TW, XC, LX, RX, PY, YZ, YY, and YX: Analyzed the results; TW and XC: Drafted the work; HC, LZ, and ML: Wrote and revised the manuscript. All authors reviewed and approved the manuscript.

### Conflict of interest statement

The authors declare that the research was conducted in the absence of any commercial or financial relationships that could be construed as a potential conflict of interest.
