# Supplementary material for: Kisspeptin Receptor GPR54 Promotes Adipocyte Differentiation and Fat Accumulation in Mice
Source: Front Physiol. 2018 Mar 13;9:209. doi: 10.3389/fphys.2018.00209 (PMC5859022; doi:10.3389/fphys.2018.00209)
Supplement: Supplementary file 1 [file Presentation1.PDF]

The Kisspeptin Receptor GPR54 Promotes Adipocyte Differentiation and Fat Accumulation in Mice

Tongtong Wang, Xueqin Cui, Ling Xie, Roumei Xing, Panpan You, Yongliang Zhao, Yiqing Yang, Yongqian Xu, Li Zeng, Huaqing Chen and Mingyao Liu

Supplementary information

Supplementary Table 1 Sequences for PCR primers

Genes	Sequences
**Gpr54**	Forward：5'-TGGTCGGAAACTCATTGGTC-3'
	Reverse：5'-AGACCTGCTGGATGTAGTTG-3'
**β-actin**	Forward：5'-GTACGCCAACACAGTGCTG-3'
	Reverse：5'-CGTCATACTCCTGCTTGCTG-3'
**Acc1**	Forward：5'-ATGGGCGGAATGGTCTCTTTC-3'
	Reverse：5'-TGGGGACCTTGTCTTCATCAT-3'
**Fas**	Forward：5'-GGAGGTGGTGATAGCCGGTAT-3'
	Reverse：5'-TGGGTAATCCATAGAGCCCAG-3'
**ADIPO**	Forward：5'-GCCAAACACCGATTGGGGT-3'
	Reverse：5'-GGCTCCAAATCTCCTTGGTAGTT-3'
**PPARγ**	Forward：5'-TCGCTGATGCACTGCCTATG-3'
	Reverse：5'-GAGAGGTCCACAGAGCTGATT-3'
**SREBP**	Forward：5'-GATGTGCGAACTGGACACAG-3’
	Reverse：5'-CATAGGGGGCGTCAAACAG-3'
**Ucp-1**	Forward：5'-ACCAAGGGCTCAGAGCATGCA-3'
	Reverse：5'-TGGCTTTCAGGAGAGTATCTTTG-3'
**IL-6**	Forward：5'-GAGGATACCACTCCCAACAGACC-3'
	Reverse：5'-AAGTGCATCATCGTTGTTCATACA-3'
**Arg1**	Forward：5´-TGGCTTGCGAGACGTAGAC-3´
	Reverse：5´-GCTCAGGTGAATCGGCCTTTT-3´
**IL-1β**	Forward：5´-GCTTCAGGCAGGCAGTATC-3´
	Reverse：5´-AGGATGGGCTCTTCTTCAAAG-3´
**CD206**	Forward：5´-CATGAGGCTTCTCCTGCTTCTG-3´
	Reverse：5´-TTGCCGTCTGAACTGAGATGG-3´
**IL-10**	Forward：5´-GCTCTTACTGACTGGCATGAG-3´
	Reverse：5´-CGCAGCTCTAGGAGCATGTG-3´
**IL-12p40**	Forward：5´- TGGGAGTACCCTGACTCCTG -3´
	Reverse：5´- GCCTTTGCATTGGACTTCGG -3´
**CCL2**	Forward：5´-TAAAAACCTGGATCGGAACCAAA-3´
	Reverse：5´- GCATTAGCTTCAGATTTACGGGT -3´
**Cidea**	Forward：5´- AGTCATCACAACTGGCCTGG -3´
	Reverse：5´- CAGCAGATTCCTTAACACGGC -3´
**CD137**	Forward：5´- GGTCAGGGGTTCTGAGTTCC -3´
	Reverse：5´- ACCTGAAATAGCCTGCACACA -3´
**DIO2**	Forward：5´- ACCCAGTTTAACCTGTTTGTAGG -3´
	Reverse：5´- ATGGGACTCCTCAGCGTAGA -3´
**Prdm16**	Forward：5´- GAACCAGGCATCCACTCGAA -3´
	Reverse：5´- TCATTGCATATGCCTCCGGG -3´
**TMEM26**	Forward：5´- GGCTACAAATGGCTTTCTCCG -3´
	Reverse：5´- GGACAGGGTCTTGCCTTGAG -3´

Supplementary Figures

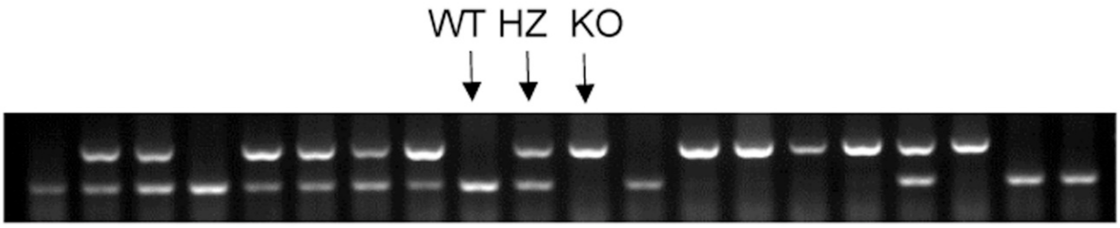

Supplementary Figure S1 Representative results of genotyping. Genotyping was preformed by PCR. WT, wild-type; HZ, *Gpr54*$^{+/-}$; KO, *Gpr54*$^{-/-}$.

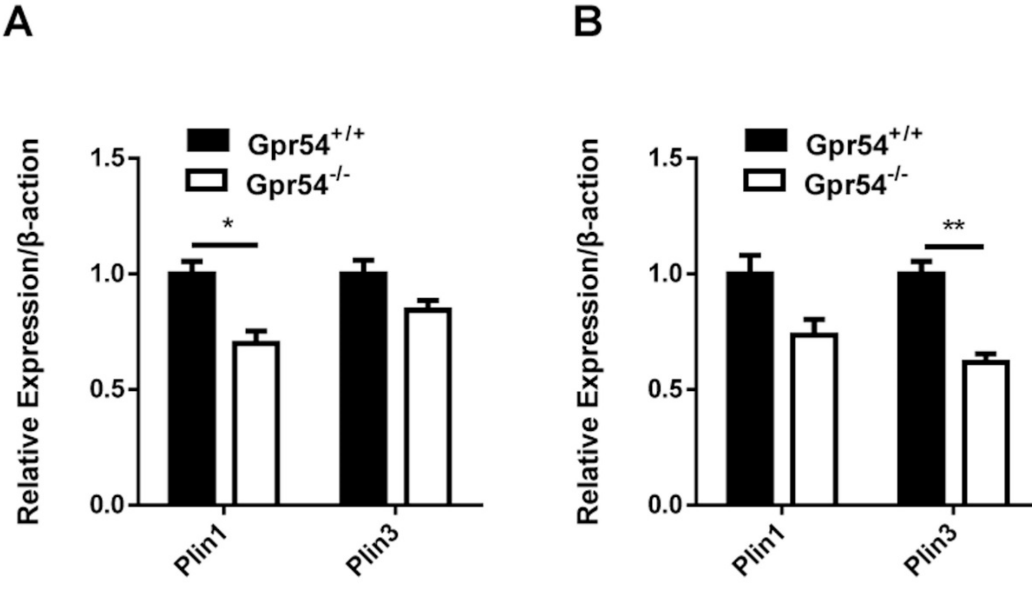

Supplementary Figure S2 Expression of perilipin. Real-time PCR analysis using samples from liver and VAT of female WT and *Gpr54*$^{-/-}$ mice fed in HFD (triplicates).
